# Combined prime-boost vaccination against tick-borne encephalitis (TBE) using a recombinant vaccinia virus and a bacterial plasmid both expressing TBE virus non-structural NS1 protein

**DOI:** 10.1186/1471-2180-5-45

**Published:** 2005-08-02

**Authors:** SE Aleshin, AV Timofeev, MV Khoretonenko, LG Zakharova, GV Pashvykina, JR Stephenson, AM Shneider, AD Altstein

**Affiliations:** 1 Chumakov Institute of Poliomyelitis and Viral Encephalitis RAMS, Moscow Region, Russia; 2 Institute of Gene Biology RAS, Moscow, Russia; 3 London School of Hygiene and Tropical Medicine, London, UK; 4 Cure Lab Inc., Stoughton, MA, USA; 5 Engelhardt Institute of Molecular Biology RAS, Vavilov str. 32, 119991 Moscow, Russia

## Abstract

**Background:**

Heterologous prime-boost immunization protocols using different gene expression systems have proven to be successful tools in protecting against various diseases in experimental animal models. The main reason for using this approach is to exploit the ability of expression cassettes to prime or boost the immune system in different ways during vaccination procedures. The purpose of the project was to study the ability of recombinant vaccinia virus (VV) and bacterial plasmid, both carrying the NS1 gene from tick-borne encephalitis (TBE) virus under the control of different promoters, to protect mice against lethal challenge using a heterologous prime-boost vaccination protocol.

**Results:**

The heterologous prime-boost vaccination protocol, using a VV recombinant and bacterial plasmid, both containing the NS1 TBE virus protein gene under the control of different promoters, achieved a high level of protection in mice against lethal challenge with a highly pathogenic TBE virus strain. No signs of pronounced TBE infection were detected in the surviving animals.

**Conclusion:**

Heterologous prime-boost vaccination protocols using recombinant VV and bacterial plasmids could be used for the development of flavivirus vaccines.

## Background

Prime-boost immunization protocols using different expression systems have proven to be successful tools in protecting experimental animals against various important human diseases [1] including tuberculosis [2], AIDS [3], and hepatitis C [4]. The success of such vaccination schemes depends upon the efficiency of the expression systems, of which, recombinant vaccinia virus(VV) and bacterial plasmid vectors are among the more common systems studied [6-8]. Moreover, it has been shown that when at least one component in a prime-boost vaccination scheme includes a plasmid vector, there is a strong response from both pathways of the host immune system [8]. There is no unique mechanism to account for the efficiency of prime-boost vaccination protocols, but it is well known that synergy of priming and boosting in the host using different expression vectors evokes high-avidity CD4 and CD8 cells [5]. Consequently, several such vaccination protocols are now in human clinical trials [1,6]. Here we present the results of experiments employing the sequential use of recombinant VV and a bacterial plasmid, both expressing non-structural protein, NS1, of tick borne encephalitis (TBE) virus, in protecting mice against lethal virus infection. It has previously been shown that both expression systems independently can partially protect mice against lethal challenge with the highly pathogenic TBE virus strain but only after repeated revaccinations [9,10]. Despite the efficiency of commercially available whole-virion TBE vaccines [11], concerns have been expressed about the development of non-neutralizing anti-envelope antibodies that could enhance the infection [12] if vaccination protocols are not followed, as well as if individuals are subclinically infected with other flaviviruses or immune compromised. The use of another protective TBE virus antigen, such as the NS1 non-structural protein, presented by different expression systems may help to address these concerns in some circumstances [9,10,13].

## Results and discussion

The results that were obtained with different vaccination protocols against TBE are presented in Table 1. The combined vaccination scheme of both vectors without the NS1 gene (VV-WR and pMV100) did not protect mice against lethal challenge (0% of protection). The recombinant vaccinia virus (W-NS1) in combination with the control plasmid pMV100 protected 20% of challenged mice, whereas 40% of mice survived that were primed with control vaccinia vector VV-WR and boosted with the NS1 gene expressing plasmid pMV45 (the difference is statistically insignificant). When mice were primed with the recombinant VV W-NS1 and boosted with plasmid pMV45, the level of protection significantly increased (80%).

**Figure 1:**
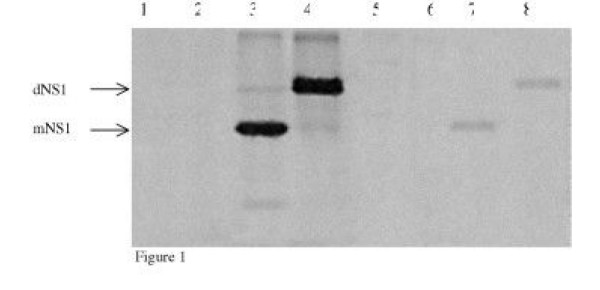
**Study of mouse sera in RIPA with the TBE virus infected and radioactive labeled SPEV cell extracts**. Lanes 1 and 2 – serum from intact mice; lanes 3 and 4 – mouse anti-NS1 sera [9]; lanes 5 and 6 – pooled sera of mice vaccinated with the vaccinia virus recombinant (W-NS1); lanes 7 and 8 – pooled sera from mice primed with W-NS1 after booster vaccination with pMV45. Odd lanes: samples were boiled before SDS electrophoresis to detect NS1-monomers; even lanes: samples were not boiled before electrophoresis to detect NS1-dimers. The position of monomers (mNS1) and dimers (dNS1) of the NS1 protein are marked with arrows.

**Figure 2:**
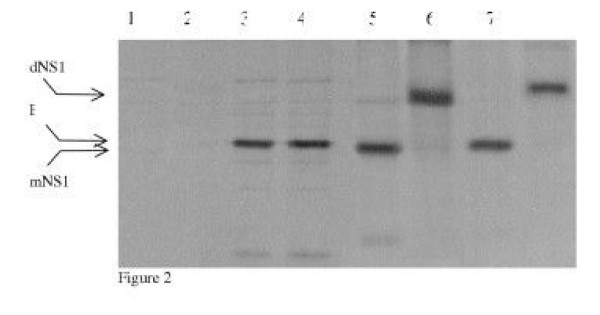
**Study of mouse sera in RIPA with the TBE virus infected and radioactive labeled SPEV cell extracts**. Lanes 1 and 2 – serum from intact mice; lanes 3 and 4 – anti-E protein Mab; lanes 5 and 6 – mouse anti-NS1 serum [9]; lanes 7 and 8 – pooled sera from mice vaccinated with W-NS1 and pMV45 which survived after the challenge with the TBE virus. Odd lanes: samples were boiled before SDS electrophoresis; even lanes: samples were not boiled before electrophoresis. The position of E protein, monomers (mNS1) and dimers (dNS1) of the NS1 protein are marked with arrows.

Detection of anti-NS1 and anti-E (envelope protein) antibodies in vaccinated and challenged mice was carried out by radioimmuno precipitation analysis (RIPA). The analysis did not detect anti-NS1 antibodies in the blood of mice after primary vaccination with the recombinant vaccinia virus (W-NS1) (Fig. 1, lanes 5 and 6) but they were detected after booster vaccination with the recombinant plasmid pMV45 (Fig. 1, lanes 7 and 8). Anti-NS1 antibodies were also detected in vaccinated animals after subsequent challenge with the TBE virus (Fig. 2, lanes 7 and 8). However, sera from surviving mice did not contain antibodies against TBE virus E protein, suggesting that pronounced virus infection was not present in vaccinated and challenged animals (Fig. 2, lanes 7 and 8). In separate experiments, neither the W-NS1 recombinant nor the pMV45 plasmid, introduced alone, induced detectable amount of anti-NS1 antibodies in mice (data not shown).)Antibodies against NS1 protein could protect mice against the lethal challenge with homologous flaviviruses including TBE virus [15,16]. Involvement of Th_1 _immune response in protection elicited by the combined vaccination with two recombinant systems used in this study can not be excluded.

These studies are the first to demonstrate that the combined use of well-characterised expression systems employing recombinant vaccinia viruses and bacterial plasmids (DNA vaccination) can protect experimental animals against a lethal flavivirus infection. Both these expression systems could be rapidly deployed to provide simple methods of constructing vaccines against several dangerous emerging diseases that have caused severe threats to public health in many different countries during the past few years.

In our experiments we used the poxvirus recombinant based on the WR laboratory strain which replicates better in cell cultures and mice than other strains of VV. Although more convenient for our experiments, this strain could not be used directly for vaccination of humans due to its relatively high virulence. However, it can easily be used to prepare the analogous recombinants on the basis of vaccinal VV strains (Lister, Copenhagen, Praha and others) by inserting the NS1 gene into their tk gene. These strains have been broadly used for immunization against smallpox in the past.

Although vaccination against smallpox was stopped many years ago, it could be reinstated if a bioterrorist attack is thought to be imminent. Indeed, the USA, UK and several other European countries have stockpiled traditional vaccines as a precaution against such an event. However, it could be argued that it is more sensible to vaccinate humans, not with the traditional vaccinal VV strains, but with their recombinants carrying genes encoding protective antigens from other pathogens, inserted into the VV thymidine kinase (*tk*) gene [17]. Despite the fact that VV *tk*^- ^recombinants have never been used for vaccinations against smallpox in the past, there are many reasons that they could protect against the disease as with the original VV strains: 1) they have the same antigenic structure; 2) a *tk*^- ^recombinant constructed on the basis of the Lister strain that expressed hepatitis B virus HBsAg antigen [18] could efficiently replicate in calf skin, which enabled the production of dermal recombinant vaccine against hepatitis B and to conduct human trials [19]; 3) this recombinant induced a specific reaction in rabbit, calf and human skin and evoked neutralizing antibodies against orthopoxviruses [19,20]. Such recombinants would have substantial advantages over traditional VV. Not only would they possess reduced neurovirulence due to destruction of the *tk *gene [18,21,22] (residual neurovirulence of VV is a main cause of severe postvaccinal complications), but they would also be able to offer protection against smallpox and another pathogen. For instance, VV recombinants expressing protective antigens of TBE virus, including NS1 protein, could be used in TBE endemic regions. In this case, prime-boost vaccination protocols, which include recombinant bacterial plasmids as a booster, could provide protection against TBE without raising concerns that pre-existing immunity to vaccinia virus could reduce the immune response to TBE viral proteins. Such strategies could also help to cut the cost of the vaccination procedure, thus making them attractive public health interventions.

## Conclusion

A combined prime-boost vaccination protocol, including a VV recombinant and a bacterial plasmid, both containing the NS1 non-structural TBE virus protein gene, gave high levels of protection in mice against lethal challenge with the highly pathogenic TBE virus strain. This prime-boost vaccination protocol could be employed in the development of novel flavivirus vaccines for use in TBE endemic areas and in situations where a reintroduction of smallpox may occur.

## Methods

### Viruses and plasmids

The TBE virus strains Absettarov and Sophyin were obtained from the collection of viruses at the Chumakov Institute of Poliomyelitis and Viral Encephalitis RAMS, as mouse brain suspensions with known LD_50 _titres. The recombinant bacterial plasmid pMV45, carrying the gene for the non-structural TBE virus protein NS1 (Western subtype Neudorfl), under the control of the cytomegalovirus immediate early promoter has been described earlier [13]. Plasmid pMV100, carrying the cytomegalovirus expression cassette without an insert was used as a negative control [13]. Plasmid preparations were performed according to EndoFree Plasmid Mega protocol (QIAGEN Inc., USA). The construction and characterisation of the VV recombinant (W-NS1), expressing the TBE virus non-structural NS1 protein gene, inserted into the *tk *gene under the control of the synthetic early-late poxvirus promoter has been described previously [10]. The recombinant and the parental vaccinia virus strain (WR strain) were propagated and titrated in the CV1 simian kidney cells.

### Animal experiments

Male Balb/c mice weighing 10–12 g were used. The VV recombinant and the negative control virus were injected intraperitoneally as doses of 10^7 ^PFU/mouse. The bacterial plasmids were introduced intramuscularly as 50 μg doses per mouse. The interval between vaccinations was 2 weeks. Two weeks after the last vaccination, mice were challenged intraperitoneally with 100 LD_50 _of the highly virulent TBE virus strain Absettarov (Western subtype) and monitored for 3 weeks. Not less then 5 mice from each group were bled before the challenge and their sera were pooled. The survivors from groups vaccinated with W-NS1 and pMV45 were also bled and their sera pooled for analysis. All experiments involving live animals were conducted according to International guidelines on the ethical use of animals.

### Analysis by radioimmune precipitation (RIPA)

Continuous cultures of porcine embryo kidney cells (SPEV) were infected with 1 PFU/cell of TBE virus (strain Sophyin). Thirty-six hours after infection Earl's 199 medium with 0.1% of human serum albumin (v/v) was substituted for Earl's salt solution. A ^14^C-labelled amino acid mixture (Amersham, USA) was added at a concentration of 2.5 μCi/ml and the cell sheets were harvested 12 hours later, lysed in immune precipitation buffer followed by three rounds of freezing and thawing as described previously [23].

The RIPA procedure was carried out for detection of antibodies against TBE virus NS1 and envelope E proteins using Protein A -Sepharose with subsequent electrophoresis of precipitates in 10% SDS-PAGE and exposure to X-ray film as described earlier [9,23]. E and NS1 protein monomers migrate closely on 10% SDS PAGE gel. In order to identify them on a gel, we used the boiled and unboiled variants of samples for electrophoresis. The unboiled samples of NS1 protein contain NS1 dimers. Boiling does not influence the electrophoretic mobility of E protein. Mouse anti-NS1 serum was obtained by vaccination of animals with pMV45 [9]. Monoclonal antibody (Mab) against TBE virus E protein was a kind gift of Dr. A. Kushch (Ivanovsky Institute of Virology RAMS, Moscow, Russia).

## Authors' contributions

**AMS **and **JRS **designed the general principles of the project, **ADA **and **AVT **were involved in design of experiments and drafting the manuscript and **SEA **and **MVK **carried out animal and immunological experiments. **LGZ **and **GVP **carried out the microbiological and virological studies and the manuscript was read and approved by all the authors.

## Figures and Tables

**Table 1:** Protection of experimental animals against TBE virus in mice immunized with a vaccinia virus recombinant and a bacterial plasmid, both carrying the gene for the NS1 non-structural TBE virus protein

Group number	Primary immunization	Booster immunization	surviving/infected animals (% of survivers ± SE)
1	VV W-NS1	pMV45	8/10 (80 ± 13%)
2	VV W-NS1	pMV100	2/10 (20 ± 13%)
3	VV WR	pMV45	4/10 (40 ± 15%)
4	VV WR	pMV100	0/10 (0%)
5	None	None	0/10 (0%)

*- Balb/c mice were primed either with vaccinia viruses WR or W-NS1, boosted with bacterial plasmids pMV45 or pMV100 and challenged intraperitoneally with the TBE virus (Absettarov strain, 100 LD_50_) as described in the Materials and Methods section. All the animals were monitored for 3 weeks after challenge with TBE virus. Standard error (SE) of a percent value was determined by the equation: SE = Square root of p × (100-p)/n, where p is a percent value and n is a number of animals used. Significance between two percent values (with probability 0.95): t = P1-P2/square root of SE1^2 ^+ SE2^2 ^≥ 2.0 [14].
